# Supplementary material for: Data-driven electron-diffraction approach reveals local short-range ordering in CrCoNi with ordering effects
Source: Nat Commun. 2022 Nov 4;13:6651. doi: 10.1038/s41467-022-34335-0 (PMC9636235; doi:10.1038/s41467-022-34335-0)
Supplement: Supplementary file 1 — Supplementary Information [file 41467_2022_34335_MOESM1_ESM.pdf]

Supplementary Materials for

Data-driven electron-diffraction approach reveals local short-range ordering in CrCoNi with ordering effects

Haw-Wen Hsiao[1,2†], Rui Feng[3†], Haoyang Ni[1,2], Ke An[3], Jonathan D. Poplawsky[4], Peter K. Liaw[5], and Jian-Min Zuo[1,2*]

[1]Department of Materials Science and Engineering, University of Illinois at Urbana-Champaign, 1304 W Green St, Urbana, IL 61801, United States
[2]Fredrick Seitz Materials Research Laboratory, University of Illinois at Urbana-Champaign, 104 S Goodwin Ave, Urbana, IL 61801, United States
[3]Neutron Scattering Division, Oak Ridge National Laboratory, Oak Ridge, TN, 37831, United States
[4]Center for Nanophase Materials Sciences, Oak Ridge National Laboratory, Oak Ridge, TN, 37831, United States
[5]Department of Materials Science and Engineering, The University of Tennessee Knoxville, Knoxville, TN 37996, United States
†First two authors contributed equally to this work

*Correspondence to: jianzuo@illinois.edu

This file includes:

Supplementary Notes 1 to 8
Supplementary Figs. S1 to S13
Supplementary References

Supplementary Notes

<u>Supplementary Note 1. Data mining of four-dimensional scanning transmission electron microscopy (4D-STEM) diffraction datasets</u>

To identify characteristic local diffuse scattering patterns, we developed the data mining approach outlined in **Fig. 2** of main text. Here we provide further details on the major steps involved in data mining.

A. Bragg reflections removal and cepstral analysis

The success of data mining is greatly helped by removing the strong transmitted beam and Bragg diffraction spots in the recorded diffraction patterns (DPs). **Supplementary Figure 3** demonstrates this improvement, using the virtual annular dark-field (ADF) images reconstructed from the collected four-dimensional diffraction data (4D-DD) of the water-quenched sample (Sample WQ) as an example. The virtual ADF image of total scattering is obtained by integrating diffraction intensity in as-recorded DPs with the inner and outer cutoff angles of 3.2 mrad and 32.1 mrad, respectively. Both Bragg reflections and diffuse scattering are included here. The ADF image reveals nanoclusters (NCs) of few nanometers (nm) in size with weak bright contrast on a non-uniform background (**Supplementary Figure 3A**). The background ADF intensity variation across the scanned area suggests changes of electron-diffraction conditions, e.g., in the crystal thickness and orientation. The ADF image constructed from diffuse scattering only (**Supplementary Figure 3B**) shows improved contrast for the NCs. However, the same background variation in **Supplementary Figure 3A** is also observed here. **Supplementary Figure 3C** presents the cepstral ADF image, where NCs are clearly observed, including these NCs that

weakly appear in **Supplementary Figures 3A&B**. The contrast enhancement and the removal of the non-uniform background in **Supplementary Figure 3C** greatly assist the detection of NCs.

We use the log method to remove the Bragg reflections and the background intensity in a region-averaged DP (the reference DP), using following equation as described in Ref. (*1*):

$$\Delta I(\vec{\mathbf{k}}) = \log[I(\vec{\mathbf{k}})] - \log[I_o(\vec{\mathbf{k}})] = \log\left[\frac{I(\vec{\mathbf{k}})}{I_0(\vec{\mathbf{k}})}\right] \tag{1}$$

where $\vec{\mathbf{k}}$ is the scattered wavevector, and $I_0(\vec{\mathbf{k}})$ represents the intensity in the reference DP, while $I(\vec{\mathbf{k}})$ is the intensity in a diffraction pattern contained in the 4D-DD. The DP, $I(\vec{\mathbf{k}})$, can be separated into two parts under the approximations that electron-diffuse scattering, $I_D(\vec{\mathbf{k}})$, is weak, as shown in **Supplementary Figure 2B**, and the wave function associated with diffuse scattering has a random phase, hence according to Ref. (*2*)

$$I(\vec{\mathbf{k}}) = I_o(\vec{\mathbf{k}}) + \bar{I}_o(\vec{\mathbf{k}}) * I_D(\vec{\mathbf{k}}) \tag{2}$$

where $\bar{I}_o(\vec{\mathbf{k}})$ is the thickness-averaged diffraction intensity. The convolution and thickness averaging reflect that there are many beams in $I_0(\vec{\mathbf{k}})$, each contributing to the total electron diffuse scattering, as the beam travels through the entire sample thickness (*2, 3*). Using

$$\log[I(\vec{\mathbf{k}})] \approx \log I_o(\vec{\mathbf{k}}) + \Lambda(\vec{\mathbf{k}}) * I_D(\vec{\mathbf{k}}) \tag{3}$$

where $\Lambda(\vec{\mathbf{k}}) = \bar{I}_o(\vec{\mathbf{k}})/I_o(\vec{\mathbf{k}})$, we have

$$\Delta I(\vec{\mathbf{k}}) \approx \Lambda(\vec{\mathbf{k}}) * I_D(\vec{\mathbf{k}}) \tag{4}$$

Thus, the difference log intensity obtained in Equation (S1) is simply the diffuse-scattering intensity convoluted by $\Lambda(\vec{\mathbf{k}})$, which in a thin sample with a strong transmitted beam and a relative constant intensity in the beam is simply the Fourier transform (FT) of the beam-shape function.

B. Cepstral analysis

Difference cepstrum (dC_p) analysis of electron-diffuse scattering is performed according to

$$dC_p(\vec{\mathbf{r}}_\mathbf{p}) = \left| FT \left\{ \log \left[\frac{I(\vec{\mathbf{k}},\vec{\mathbf{r}}_\mathbf{p})}{I_0(\vec{\mathbf{k}})} \right] \right\} \right| \tag{5}$$

at the probe position, $\vec{\mathbf{r}}_\mathbf{p}$. The interpretation of dC_p is made based on the separation of the fluctuating part of the scattering potential (U_1) from the average scattering potential (U_o). The scattering potential seen by a nanosized electron beam is thus

$$U(\vec{\mathbf{r}},\vec{\mathbf{r}}_\mathbf{p}) = U_o(\vec{\mathbf{r}}) + U_1(\vec{\mathbf{r}},\vec{\mathbf{r}}_\mathbf{p}) \tag{6}$$

where $U_1(\vec{\mathbf{r}},\vec{\mathbf{r}}_\mathbf{p})$ varies with the electron-probe position, $\vec{\mathbf{r}}_\mathbf{p}$. Diffraction by $U(\vec{\mathbf{r}},\vec{\mathbf{r}}_\mathbf{p})$ gives the local diffraction pattern, $I(\vec{\mathbf{k}},\vec{\mathbf{r}}_\mathbf{p})$, while diffraction by U_o gives $I(\vec{\mathbf{k}})$. For example, a random alloy would yield $\langle U_1(\vec{\mathbf{r}},\vec{\mathbf{r}}_\mathbf{p})\rangle_{\vec{\mathbf{r}}_\mathbf{p}} = 0$. According to Shao et al. (*1*), dC_p gives the Patterson function of the fluctuating scattering potential multiplied by a shape function.

Supplementary Figure 4 shows an example of Bragg reflection removal and difference cepstrum analysis for two local areas, 1 and 2, as marked in **Supplementary Figure 3C**. These two areas show strong and weak contrast in the cepstral ADF image. The log difference DPs shown in **Supplementary Figure 4A,B** are obtained by averaging $\log\left[\frac{I(\vec{\mathbf{k}},\vec{\mathbf{r}}_\mathbf{p})}{I_0(\vec{\mathbf{k}})}\right]$ over the marked box regions, respectively. As these examples show, the log method effectively removes the strong Bragg reflections in the recorded DPs, while the cepstrum detects harmonic signals in the diffuse scattering.

C. Identification of nanoclusters

A peak finding algorithm is used to locate NCs above an intensity threshold in the cepstral ADF image. First, the intensity (I) of the image is normalized with the maximum equals to 1. At each image pixel (i,j) above an entered threshold, its intensity is compared with the intensities of neighboring eight pixels. A peak is detected if the pixel intensity is above the values of its neighbors. In the analysis reported in the main text, we used the threshold of $I_{\text{threshold}}$ of ~ 0.3 for peak finding. The size of the nanocluster was initially set at 4 nm (4 pixels in diameter). The size and peak position were then adjusted manually. For this step, the detected peaks were first marked on the image using annotations in DigitalMicrograph (https://www.gatan.com/products/tem-analysis/gatan-microscopy-suite-software) and the adjustments were made on the size and position of annotations.

D. Nanocluster diffraction patterns and grouping

After locating the n_c number of NCs in the cepstral ADF image, we average the diffuse-scattering patterns within each NC to obtain the cluster-averaged DPs. This step produced a DP stack of $\overline{\Delta I_\iota}(\vec{\mathbf{k}})$, $i = 1 \dots n_c$. Further grouping can be performed on the DP stack, using the template-matching method described in Ref. (*4*). Briefly, a template is randomly selected from the DP stack and assigned a group number. The template is then compared with other unassigned DPs in the stack, using the correlation coefficient (C_C),

$$C_C = \frac{\sum_{x,y}\{[I_A(x,y)-\overline{I_A}]\cdot[I_B(x,y)-\overline{I_B}]\}}{\sqrt{\left\{\sum_{x,y}[I_A(x,y)-\overline{I_A}]^2\right\}\cdot\left\{\sum_{x,y}[I_B(x,y)-\overline{I_B}]^2\right\}}} \qquad (7)$$

where $I_A(x,y)$ and $I_B(x,y)$ are intensities of the pixel (x,y) in the DP A and B, and $\overline{I_A}$ and $\overline{I_B}$ are mean intensities of DP A and B, respectively. DP B is taken as the diffraction template, while DP A is one of DPs in the stack. DPs with CC above a threshold value (~ 0.1 for diffuse patterns) are

placed in the assigned group. This process can be repeated, and the DPs already assigned to a group are removed from subsequent template search. The DPs belong to the same group are then averaged to yield the group-averaged DPs.

The grouping can be further iterated by taking the newly obtained group-averaged DPs as the new templates. For each DP in the stack, its C_C value with each template is calculated, and the DP is assigned to the group with the highest C_C value. The modified groups are then used to obtain the new group-averaged DPs. This process can be repeated 2 to 3 times.

The above grouping procedure is similar to the *k*-means method used in the data science for clustering, which is one of the simplest and popular unsupervised machine learning algorithms (*4*). The difference is that instead of looking for a fixed number (*k*) of clusters in a dataset, we use a C_C threshold and random search to provide an initial set of DP groups to start the clustering process.

E. Identification of diffuse diffraction patterns and surface oxides

The cluster diffuse patterns can come from surface oxides, secondary phases, as well as CSRO. A majority of NCs seen in **Supplementary Figure 3C** are surface oxides. These nano-sized oxides are too small to be seen in selected area electron diffraction (SAED, **Fig. 1c&e**), but because of the enhanced diffraction sensitivity in energy-filtered scanning electron nanodiffraction (EF-SEND), they are detected together with CSRO.

To identify diffuse scattering DPs belonging to oxides, we performed EF-SAED on the samples that were exposed to air after 30 days using a Hitachi H9500 TEM equipped with a Gatan imaging filter (GIF). The DP shows continuous rings in this case, which are indexed using the NiO {111}, {200} and {220} reflections. These rings were not initially observed in our freshly prepared

TEM samples, as demonstrated by EF-SAED in **Fig. 1c&e** and the average SEND pattern in **Supplementary Figure 2A**. **Supplementary Figure 5** identifies the surface oxide NCs. Using the indexed oxide reflection rings in **Supplementary Figure 5A**, we then identified the surface oxides among the cluster averaged DPs. The four DPs in **Supplementary Figure 5C** are cluster averaged DPs for NCs marked as 1 to 4 in **Supplementary Figure 5B.** This result demonstrates that the popular NC imaging using diffuse intensity in dark-field TEM (*5*) is not sufficiently discriminative for the detection of CSRO, and it is critical to exclude the surface oxide contribution from diffuse scattering analysis, which is only possible by spatially separating the oxide nanoparticles and the CSRO strengthened NCs in a collected 4D-DD.

Once DPs belonging to surface oxides were identified and excluded, we manually select distinct DPs with diffuse scattering centered between Bragg diffraction spots and the most symmetric patterns are used as the staring templates for the detection of CSRO.

F. Correlation imaging and the measurement of electron-diffuse scattering

To measure electron-diffuse scattering from CSRO-strengthened NCs, we follow the procedure illustrated in **Fig. 2** of the main text. First, three diffraction templates belonging to CSRO are created from the cluster-averaged log difference DPs using procedures described in (*C*), (*D*), and (*E*). These templates are then used to obtain the correlation images, employing the correlation coefficient defined in Eq. (7). The value of C_C is then used to form the correlation image. The value of C_C ranges from - 1 to 1 with C_C = 1, indicating complete similarity. From the correlation image, we then identify NCs having C_C values to be equal to or greater than a fixed, pre-defined, threshold value (~ 0.1). DPs from these selected NCs are subsequently averaged to

generate new diffraction templates. This process is repeated about two to three times to refine the diffraction templates.

Supplementary Note 2. Strain mapping

Strain analysis via SEND is based on Bragg's law by measuring the change in the distance between diffracted beams. The beam in a diffraction pattern appears as a disk because of the beam's semi-convergence angle (1.1 mrad for SEND). For each diffraction pattern, the positions of 9 selected beams, which include the center beam and 8 diffracted beams of low-order reflections, were determined, using the circular Hough transform method described by Yuan et al. (*6*). The method works by applying first the Sobel filter to the SEND pattern to filter out the disk edge. Then, circular Hough transform is applied on the filtered SEND pattern, which transforms the filtered pattern of edge circles into a pattern of Hough transformation peaks, with each peak marking the position of a detected circle. The peak position was measured by fitting, using a Lorentzian peak model. This measurement was applied to all 9 selected beams, and a 2D reciprocal lattice with $\overrightarrow{\mathbf{G_1}}$ = (002) and $\overrightarrow{\mathbf{G_2}}$ = (2-20), as the basis vectors was then determined from the measured disk positions. The calibration was performed, using a standard Si specimen. **Supplementary Figure 6** shows the obtained ε_{xx}, ε_{yy}, and ε_{xy} strain maps, plus the rotation maps, for Samples WQ and HT, respectively.

Supplementary Note 3. Diffraction simulation using first-principles theory models

The atomistic models published by Walsh et al. (*7*) were examined by diffraction simulations for comparison with our experimental DPs. All atomic configurations considered here belong to the fcc crystal with 108 atoms in a volume with 3 x 3 x 3 unit-cells. The configurations have a

range of compositions from the stoichiometric CrCoNi to off-stoichiometric compositions. The Warren-Cowley (WC) SRO parameters were used to generate the atomic configurations.

Supplementary Figure 7 summarizes the atomistic models that we included in our diffraction simulation. For the Structure 0.5 model, the following WC SRO parameters are used: $\alpha_{CrCr} = 0.5$, $\alpha_{CrCo} = -0.25, \alpha_{CrNi} = -0.25$, $\alpha_{NiCo} = 0.25$, $\alpha_{CoCo} = 0.0$, and $\alpha_{NiNi} = 0.0$. $\alpha_{ij} = 0$ is used for the quasi-random models. The WC SRO parameters for the Tamm and Ding models (*8*) were obtained from the Monte Carlo optimization of on-lattice DFT simulations (*8, 9*). In the maximum spin (Max Spin) model (*7*), the placements of Co and Cr↑Cr↑ are optimized simultaneously, fully segregating these species.

The simulated DPs in **Supplementary Figure 7** are the sum of DPs obtained from different atomic configurations in the same model. Diffuse-intensity peaks near the special positions of (100) and (110) are observed in Tamm, Structure 0.5, Max Spin and spin ordered (not shown) models. The diffuse intensity is stronger in the off-stoichiometry Structure 0.5 model than in the stoichiometric Tamm model, while the diffuse peak feature in the Max Spin model is inconsistent with the experiment.

Supplementary Figure 8 examines the fluctuations within the off-stoichiometry structure 0.5 and quasi random atomistic models as seen by diffraction. Each configuration contains 3x3x3 unit cells and approximately 1 nm^3 in volume.

Next, we performed diffraction simulations along the [112] zone axis orientation (**Supplementary Figure 9**). This zone axis was recently identified and employed for the study of CSRO in CrCoNi by Zhou et al. (*10*). In their work, a diffuse spot at the $(\bar{3}11)/2$ position was identified and attributed to CSRO. The question in hand is whether the CSRO predicted by the

structure 0.5 model also reproduces this observation. The simulation results for the off-stoichiometry quasi-random and structure 0.5 models show that diffuse scattering occurred at (1-10) position in the structure 0.5 model, while no feature is observed in the quasi-random model. Neither of these models produce visible a diffuse spot at $(\bar{3}11)/2$.

Supplementary Note 4. Estimation of Warren-Cowley short-range order (SRO) Parameter:

The SRO parameter can be determined from the Patterson function obtained from the recorded diffuse-scattering patterns, as shown in **Figs. 4d,e** of the main text. Following Cowley (*11*), the SRO parameter, α_{ij}, relates the elemental compositions, m_A and m_B, between sites, i and j, by

$$\alpha_{ij} = 1 - \frac{P_{ij}^{AB}}{m_A m_B} \tag{8}$$

where P_{ij}^{AB} is the probability of finding a B atom at a site, j, from an A atom at site, i.

Under the kinematical diffraction condition and in the absence of the size effect, the diffuse-scattering intensity (I_d) of a disordered binary alloy is given by following equation, as a function of the reciprocal space coordinate, $\vec{\mathbf{u}}$

$$I_d(\vec{\mathbf{u}}) = N m_A m_B (f_A - f_B)^2 \sum_i \alpha_{0i} \exp(2\pi i\, \vec{\mathbf{u}} \cdot \vec{\mathbf{r}}_\mathbf{i}) \tag{9}$$

where the index, i, is over the atomic sites, $\vec{\mathbf{r}}_\mathbf{i}$ (*12*). The Patterson function of the diffuse scattering $[P(\vec{\mathbf{r}})]$ can be derived as

$$P(\vec{\mathbf{r}}) = N m_A m_B \sum_i [\alpha_{0i}\{(\rho_A - \rho_B) * (\rho_A - \rho_B)\}] * \delta(\vec{\mathbf{r}} - \vec{\mathbf{r}}_\mathbf{i}) \tag{10}$$

where the ρ_A and ρ_B represent the atomic potentials of elements, A and B, respectively. The first term ($r_0 = 0$) in the Eq. (S10) yields the zero-order peak at the center of the Patterson function map, and the second and high order terms correspond to the Patterson peaks for the first order

(nearest) and higher order inter-distances between the lattice sites. The scattering potential of transition metals, ρ, is more localized than the lattice-site distances. Hence, the scattering potential at the j-th site contributes very little to the i-th site. Therefore, the diffuse scattering Patterson peak value at a specific site, i, can be written as

$$P(\vec{\mathbf{r}}_{\mathbf{i}}) \propto \alpha_{0i}. \tag{11}$$

Thus, α_{0i} at a specific site, i, can be directly obtained from a Patterson function value $P(\vec{\mathbf{r}}_{\mathbf{i}})$ in a binary alloy.

The Fourier transform of the 2D diffuse-scattering pattern gives the projected Patterson function (**Figs. 4d**,**e**, the main text). The interpretation of these patterns can be made by approximating CrCoNi as a pseudo-binary alloy of CrX_2, with X = Co or Ni, because of the small difference between the scattering potentials of Co and Ni. Using this approximation, we find $\alpha_{01} = -0.18$ and $\alpha_{02} = 0.38$ for the nearest and second nearest neighbor SRO parameter in the $L1_2$ CSRO, respectively. The estimated α_{01} is very close to the values of α_{CrCo} and α_{CrNi} reported by Tamm et al. (*8*) and Ding et al. (*9*).

Supplementary Note 5. Chemical composition

The STEM/EDX technique was employed to determine the chemical homogeneity in CoCrNi. To avoid the zone-axis electron-channeling effects (*13, 14*), we used the off-zone axis orientation (tilted ~ 2° away) to collect the EDS spectra over the areas of 100 x 100 nm^2 at a 1-nm step size. An electron probe of 1.2 nm in the full-width-half maximum (FWHM) was used for the analysis. The sample thickness in both cases was determined as ~ 50 nm by convergent-beam electron diffraction (CBED). The EDS spectra were acquired, using a four-quadrant Super-X detector (Thermo-Fisher Scientific) with the acquisition time of 1s per spectrum. The beam current was

kept at 150 pA for both samples. The K-α peaks in each X-ray spectrum were used to calculate the atomic percentage of each element. **Supplementary Figure 10** shows the elemental-chemical maps for Cr, Co, and Ni in Samples WQ and HT, respectively. The mean compositions of Cr, Co, and Ni are determined at 33.86 atomic percent (at%), 33.27 at%, and 32.87 at% in Sample WQ, and 33.50 at%, 33.89 at%, and 32.62 at% in Sample HT, respectively. Both are close to the targeted composition.

Supplementary Note 6. Guinier analysis of small-angle neutron scattering

Guinier analysis allows the model-free determination of the radius of gyration (R_g) of a scattering object from small-angle X-ray or neutron scattering data (*15*). Guinier analysis is based on a series expansion of a scattering intensity, leading to following expression

$$\ln[I(Q)] = \ln[I_0] - (R_g^2/3)Q^2, \tag{12}$$

in which Q is the scattering vector, $I(Q)$ is the measured small-angle neutron scattering (SANS) intensity, I_0 is the intensity when $Q = 0$. R_g is the radius of gyration, which represents the effective size of the scattering object. If the scattering objects have a sphere shape, the radius of the particles (R) can be determined from

$$R_g^2 = \frac{3}{5}R^2 \tag{13}$$

Linear (Guinier) scaling suggests that the system is essentially monodisperse.

Supplementary Note 7. Calculation of radius distribution functions (RDFs) from the APT data and their interpretation

We performed partial radial distribution function (RDF) analyses of APT data to assess chemical clustering within the probed APT volumes. An APT dataset is a labelled point cloud with

approximate atom positions defined in the Cartesian coordinates. A distribution function can be generated computationally within the dataset without the need of Fourier transform, as done in diffraction. The partial RDF is an average radial composition profile from every pair of atoms of interest (such as Cr-Cr) in the APT dataset (*16*). The partial RDF for *n* atoms is calculated via the summation of the inter-atomic pair distances between their respective positions, P_i and P_j, giving the distance, *r* (*17*).

$$\mathrm{RDF}(r) = \frac{1}{\bar{\rho} r^2} \sum_{i=1}^{n} \sum_{\substack{j=1 \\ j \neq 1}}^{n} \mathrm{Hist}(\| \vec{\mathbf{P}}_\mathbf{i} - \vec{\mathbf{P}}_\mathbf{j} \|) \quad (14)$$

The partial RDF is a histogram of unit impulse functions, $\delta(x - r)$, of relative atomic positions, $\vec{\mathbf{P}}_\mathbf{i}$ and $\vec{\mathbf{P}}_\mathbf{j}$, are a pair of atoms with the selected atom, *i*, at the center and another selected atom, *j*, on the surface of a sphere of a radius, *r*, in a data set of average density, $\bar{\rho}$. The summation generates a total of $n(n - 1)/2$ pairs for *n* atoms, all of which must be calculated and separated to provide the partial RDF. The interval for the RDF calculation was set at 0.5 nm, which is close to our APT resolution.

The Co, Ni, and Cr centered RDFs are displayed in **Fig. 5** for the heat-treated and water-quenched samples. These partial RDFs are averaged over the APT volume that can be considered as being made of a matrix of random solutions mixed with elemental-rich or poor NCs. This complexity makes the RDF interpretation difficult. Nonetheless, the measurements show the strongest RDF signals in the Ni-Ni, Co-Cr, and Ni-Cr RDFs and their deviation from the random solution. The net positive Ni-Ni RDF, which is stronger than the Co-Co and Cr-Cr RDFs in **Fig. 5**, can be taken as that there are more Ni-rich NCs than Ni-poor ones in a matrix, which is slightly Ni poor. The negative Co-Cr and Ni-Cr RDFs, extending to ~ 1 nm for Co- or Ni-centered atoms,

indicate the presence of Cr-poor but Ni-, Co-, or (Ni, Co)-rich clusters. In comparison, the averaged Cr-Cr is close to the matrix composition. Its difference with the Ni-Ni RDF, especially, can be attributed to the similar number of Cr-rich and poor regions and the different roles of Cr and Ni in atomic diffusion. The Ni-Ni RDF indicates that Ni-rich clusters extend to several nm, while in Co-Ni rich clusters, Co-Ni RDF is above 1 nm up to several nm. The presence of Co-Ni clustering in **Fig. 5a** and the absence of Ni-Co clustering in **Fig. 5b** is most likely due to Co being present within the Ni clusters and the stronger Ni-Ni clustering, compared to Co-Co clustering.

We note that our experimental results focus on compositional fluctuations averaged over a few shells around each detected atom and the APT data does not resolve the crystal lattice (**Fig. 5**). To detect CSRO using APT, high-resolution APT is needed to examine the correlation in first several shells around each atom. This experiment has been performed by Inoue et al. (*18*) through the APT analysis of [100]- and [111]-oriented sharp needles in small volumes. Their one-dimensional spatial distribution maps show that the elemental density along the [001] direction is modulated with Cr-rich {100} atomic layers and (Ni + Co)-rich {100} layers aligned in the FCC structure, which is consistent with $L1_2$ CSRO. Compared to these results, the APT experiments presented here allows an examination of chemical fluctuations at 0.5 nm to tens of nm length scale.

The RDFs obtained from APT are qualitative for following reasons. First, APT only detects part of atoms. Second, both the number of detected atoms and their positions depend on the field-evaporation process (*19*). For example, the *z* position is determined more precisely than the x and y positions due to the difference between distinguishing the sequence of electrical field-evaporated ions, and determining the positions using ion trajectory, with latter being subjected to aberrations associated with the electrical field evaporation. Third, the field-evaporation strengths of the

elements may vary in a multicomponent alloy. According to Hatzoglou et al. (*20*), Cr has a lower evaporation voltage than Ni. The atom maps exhibiting aberrations in the atom positions can result from the nonuniform field evaporation. Additionally, field evaporation can be impacted by the presence of vacancies or interstitials. A previous APT study of irradiated NiCoCr alloys found the Ni and Co enrichment and Cr depletion around He bubbles, which was attributed to diffusion mechanisms, e.g., Ni and Co sites facilitate interstitial diffusion, while Cr sites facilitate vacancy diffusion (*21*).

The qualitative RDF results here suggest that Ni and Co have affinity with each other, while Ni/Co and Cr tend to repel each other. The Ni and Ni/Co rich clusters are a few nm in radius. Together, they demonstrate that the chemical composition in the prepared CrCoNi alloys, regardless of the processing conditions, is heterogeneous at atomic to nm length scales.

Supplementary Note 8. Detection of CSRO by atomic-resolution high-angle annular dark-field scanning transmission electron microscopy (HAADF-STEM)

Aberration corrected high-angle annular dark-field STEM (HAADF-STEM) in principle can detect local chemical ordering (*21-23*). **Figures 1d** and **f** are two HAADF-STEM images recorded from Samples WQ and HT, respectively. Both images show rather uniform contrast at atomic resolution without indications of chemical ordering by visual inspection. This result is consistent with the report by Sales et al. (*24*). Interestingly, the power spectra (insets in **Figs. 1d and f**) of two images indicate subtle differences between the two images with the streaking diffuse intensity along {111} directions being observed in both samples. The reason why SRO is not directly observable is because of the small Z difference among Cr, Co, and Ni, which results in a small

difference in the HAADF-STEM atomic column intensities. Another reason is the noise in the recorded image, which further obscures the small intensity difference.

Fourier filtering using diffuse signals was used previously to detect SRO (*5*). A main drawback of this method is the reduced resolution, from atomic columns in the recorded images to lattice fringes in the processed images. Another drawback is the possible artefacts due to the imposition of the Fourier filtering mask. To solve these issues, we performed further HAADF-STEM imaging at the magnification of 3.6 M to cover a larger field of view than **Figs. 1d** and **f**. The images were acquired in ten frames with 0.0247 nm per pixel resolution and in a short dwell time of 1 μs with a probe current of 30 pA. The probe was aberration corrected with a semi-convergence angle of 21.4 mrad, and the detector inner cutoff angle is 40 mrad. These frames are then aligned and averaged to reduce the scan noise, as well as intensity noise in individual images. The highest frequency resolved by the power spectrum is ~ 1 Å in the averaged image.

Second, the averaged image was analyzed, using cross-correlation analysis through template matching in a two-steps process. First, a template of the atomic column is extracted from the image and then spatially averaged, employing the method described in Ref. (*25*). Second, the spatially averaged template is used to calculate the cross-correlation coefficient, as defined below

$$\mathrm{X}_C\left(r,s\right)=\frac{\sum\limits_{(i,j)\in R} I\left(r+i,s+j\right)T\left(i,j\right)}{\sum\limits_{(i,j)\in R} T\left(i,j\right)} \tag{15}$$

This method measures sensitively the degree to which an atomic column deviates from the average (**Supplementary Figure 13**). Using this method, we can detect SRO in Sample WQ, as shown in **Fig. 8** of the main text.

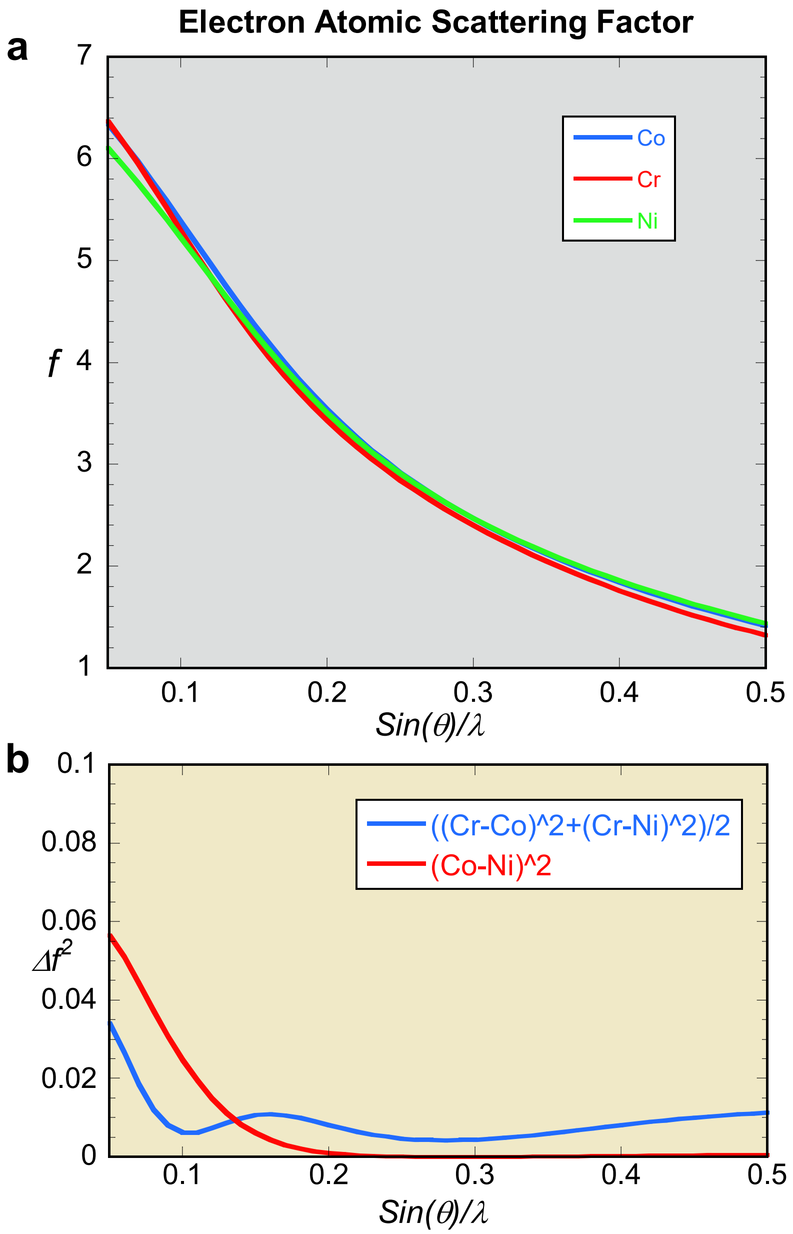

Supplementary Figure 1. A comparison of atomic-scattering factors in CrCoNi. (**a**) Atomic-scattering factors of Co, Cr, and Ni, and (**b**) their square differences.

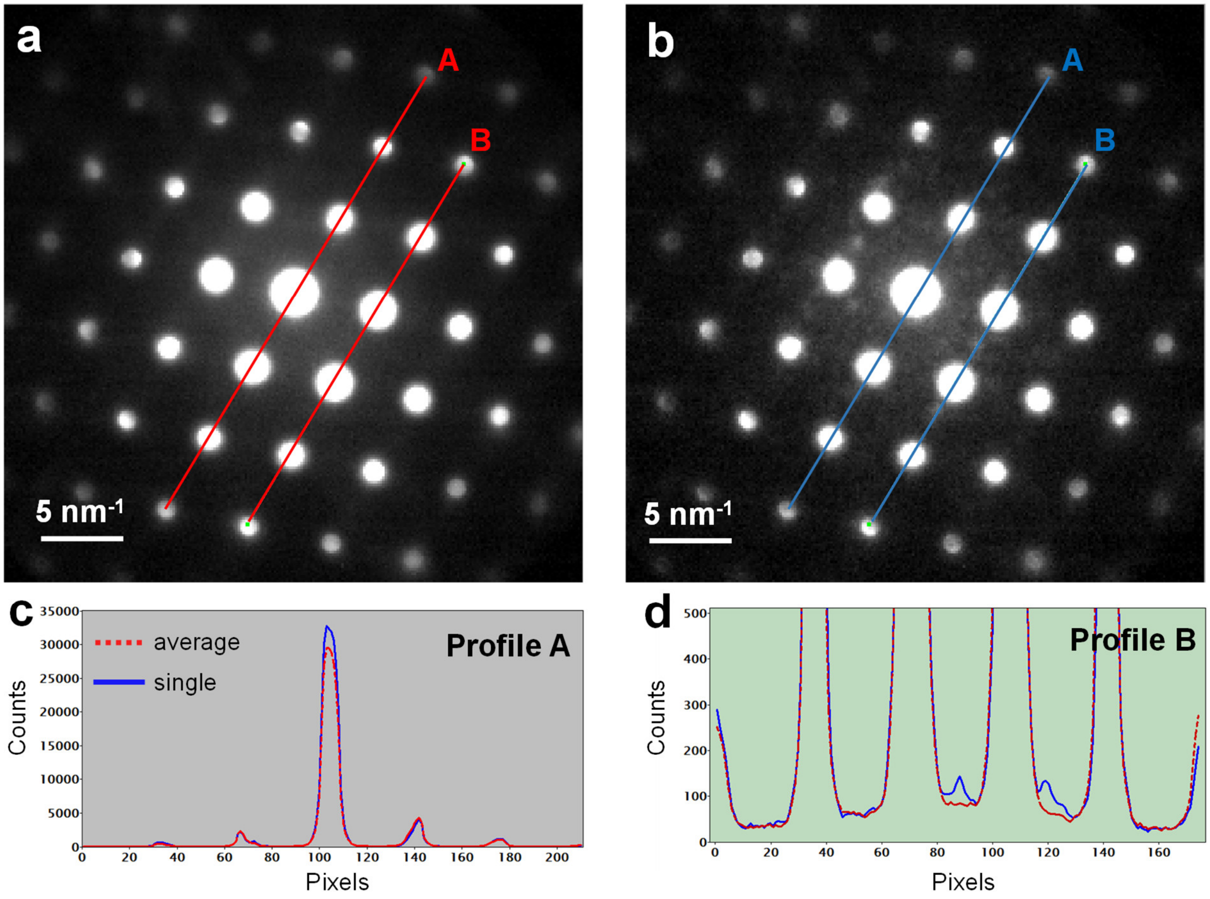

Supplementary Figure 2. The collected DP dataset from Sample HT. (**a**) the averaged DP from ten-thousand DPs, (**b**) a single DP showing local diffuse scattering, (**c**) and (**d**) intensity profiles from lines, A and B, marked in (**a**) and (**b**). The DPs are along the [110] zone axis.

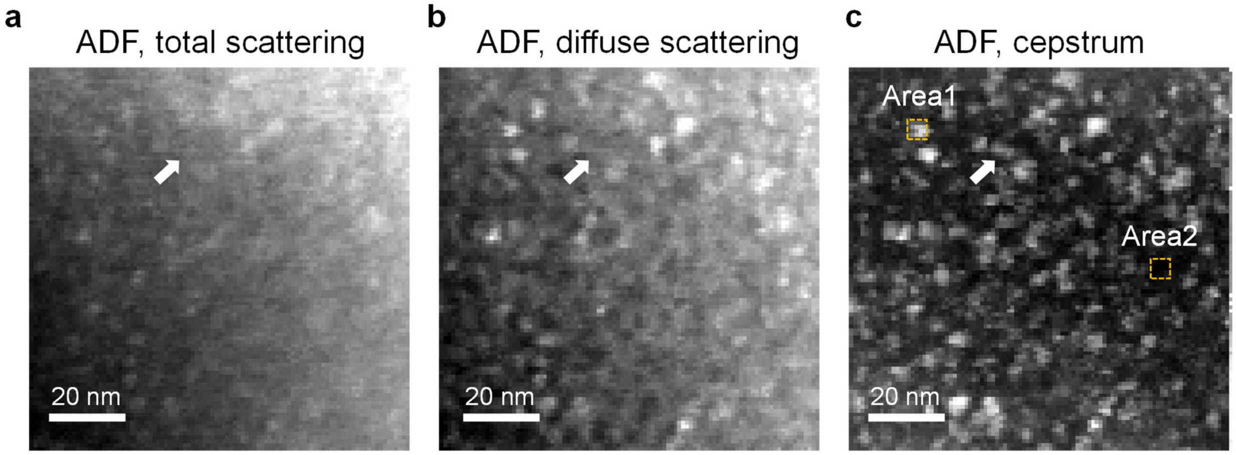

Supplementary Figure 3. Annular dark-field (ADF) images reconstructed from the 4D diffraction dataset. For Sample WQ, the reconstruction is carried out, using (**a**) total-scattering intensity, (**b**) diffuse-scattering intensity, and (**c**) diffuse-scattering intensity after cepstral transformation. The arrows mark an example of a weak NC detection by the three imaging methods.

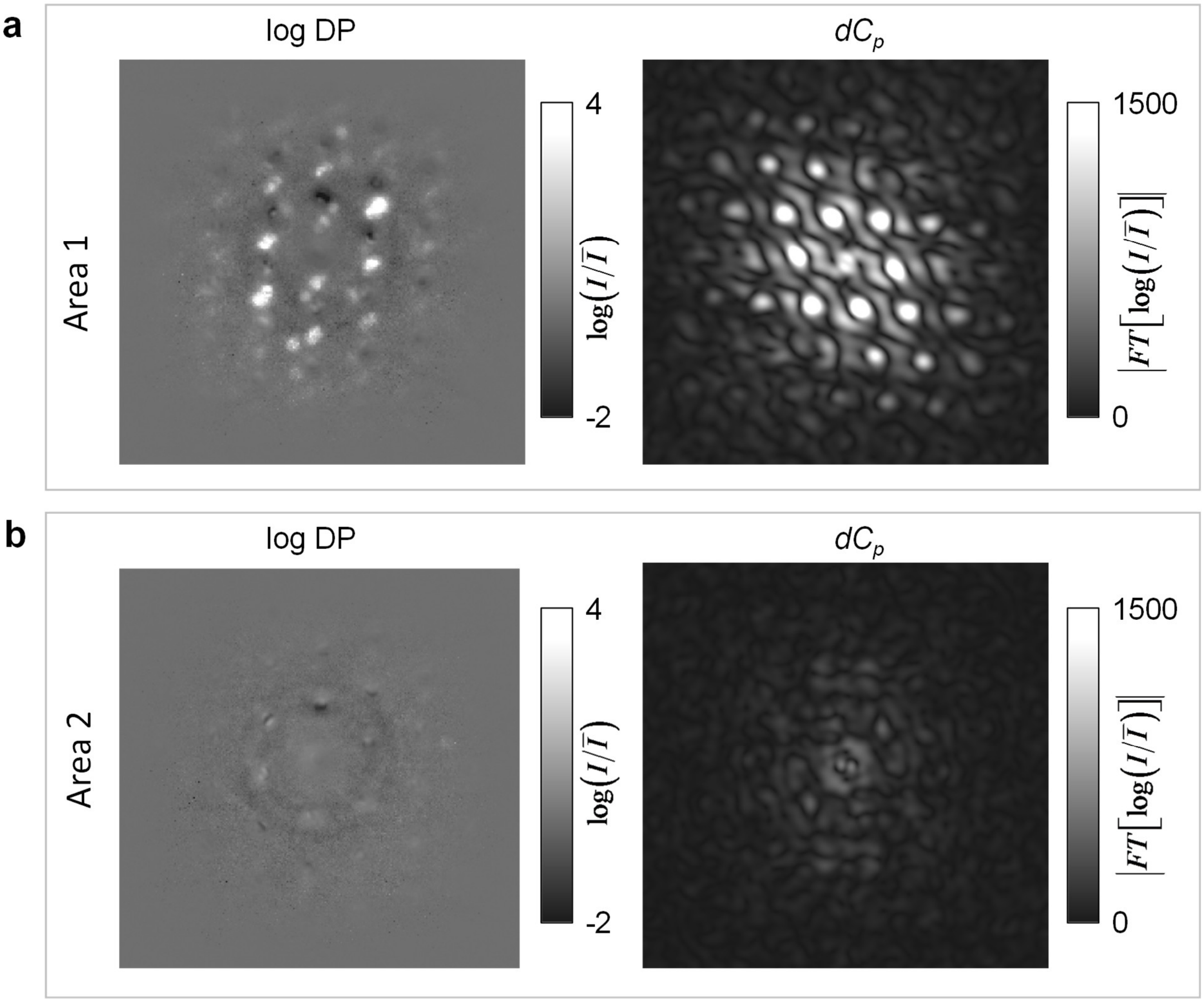

Supplementary Figure 4. Two examples of Bragg reflection removal and difference cepstrum analysis. (**a**) Area1 and (**b**) Area2 in Supplementary Figure 3. Here log DP refers to log difference DP, as given in Equation (1). The grey scale bars indicate the range of displayed numerical values.

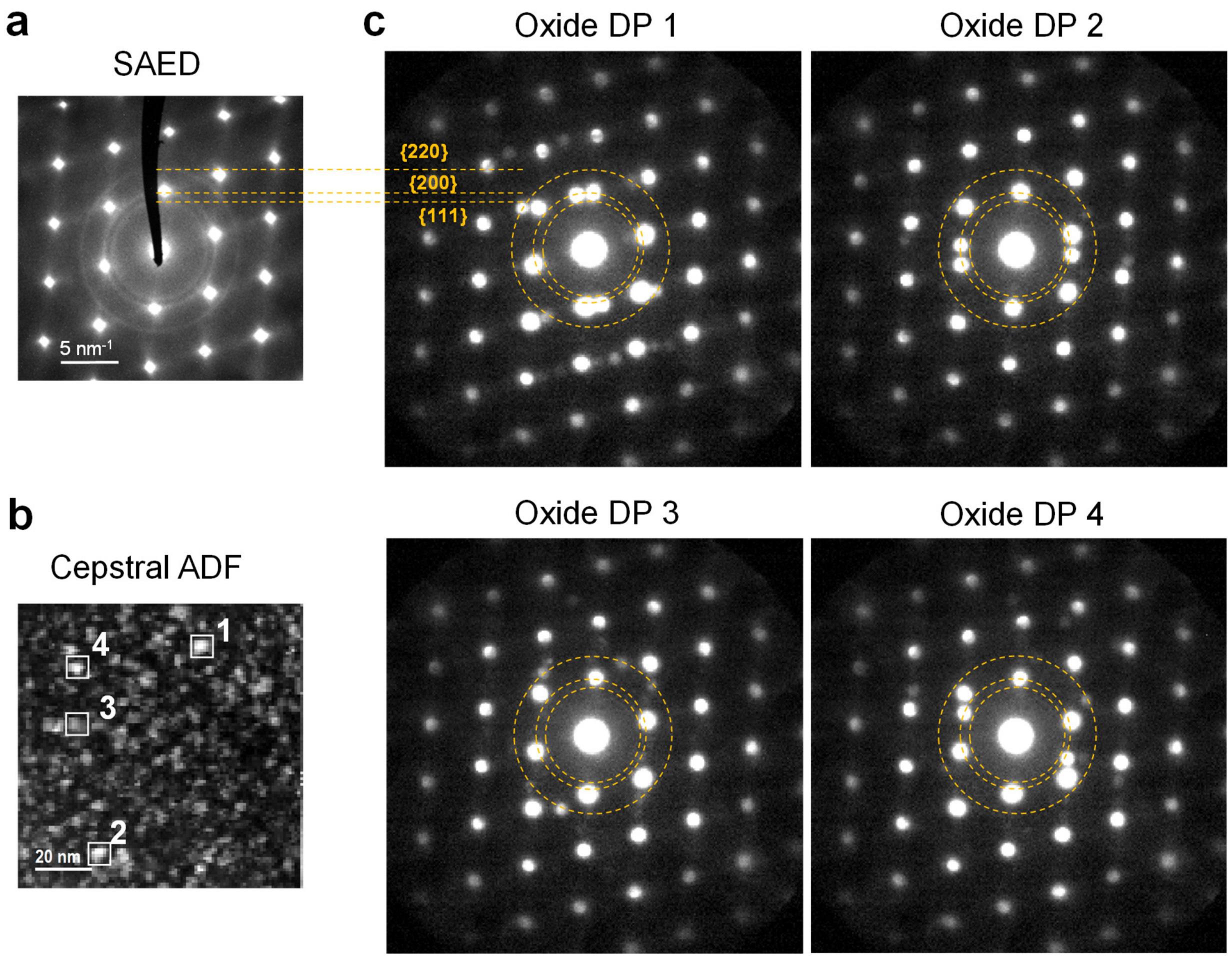

Supplementary Figure 5. Identification of surface oxides in electron-diffraction patterns. (**a**) EF-SAED pattern recorded from Sample WQ after the extended exposure to air showing the powder rings from surface oxides with the NiO structure. (**b**) A cepstral ADF image with marked surface-oxide particles. (**c**) Oxide-diffraction patterns corresponding to the four marked oxide particles in (**b**) with extra reflections falling on the rings.

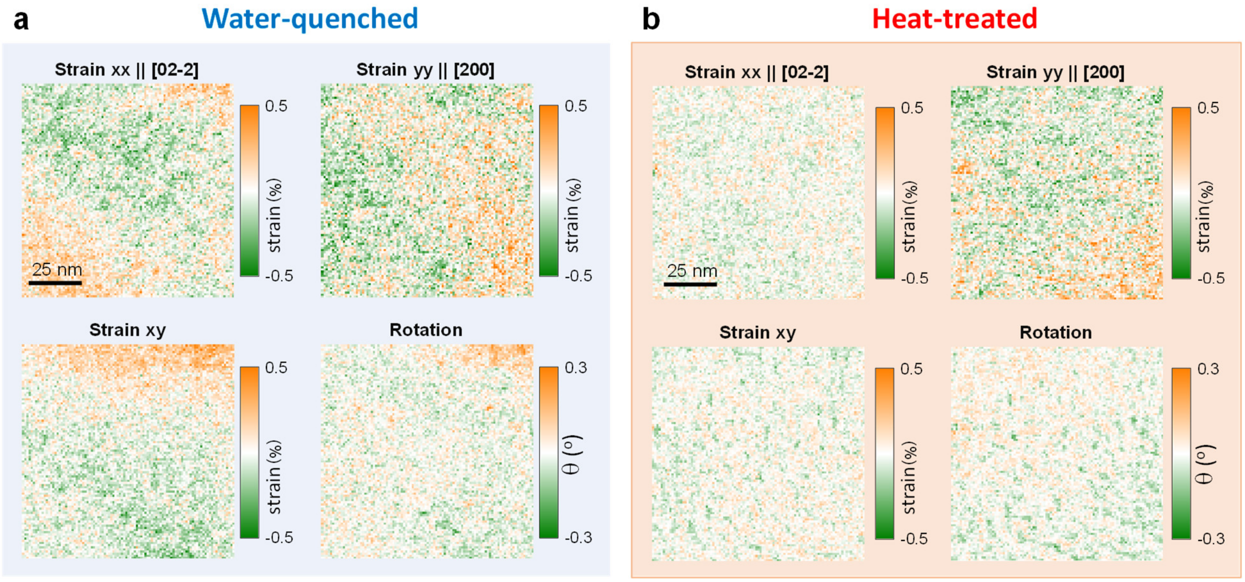

Supplementary Figure 6. Inhomogeneous distribution of strain in the water-quenched and heat-treated CrCoNi samples. The measured 2D strain with x || [02-2] and y || [200] directions projected along the [011] zone axis for (**a**) Sample WQ and (**b**) Sample HT.

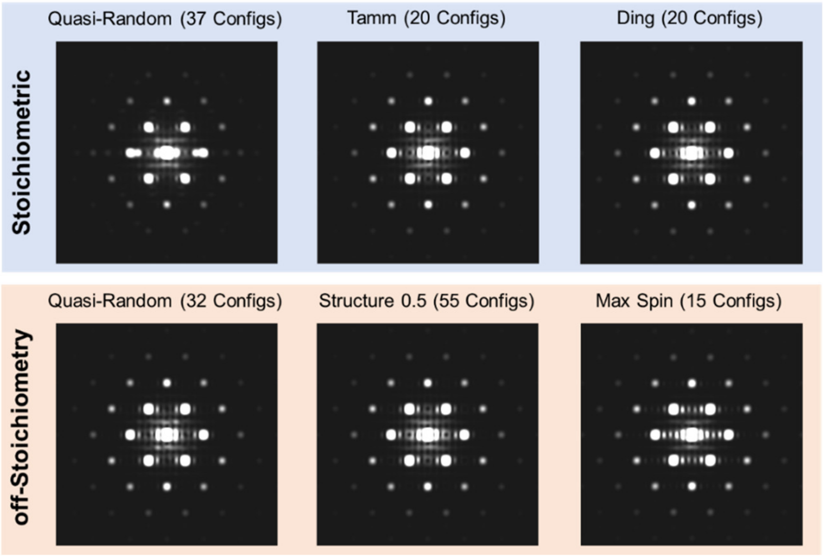

Supplementary Figure 7. Configurations-averaged [110] zone axis diffraction patterns. The patterns are calculated with the atomistic models based on the WC SRO parameters for Tamm, Ding, and Structure 0.5, and zero SRO parameters for the quasi-random models (*7*), respectively.

The Max Spin model optimizes atomic pairing for spin ordering (*7*). The number of atomistic configurations (configs) for each DP is indicated.

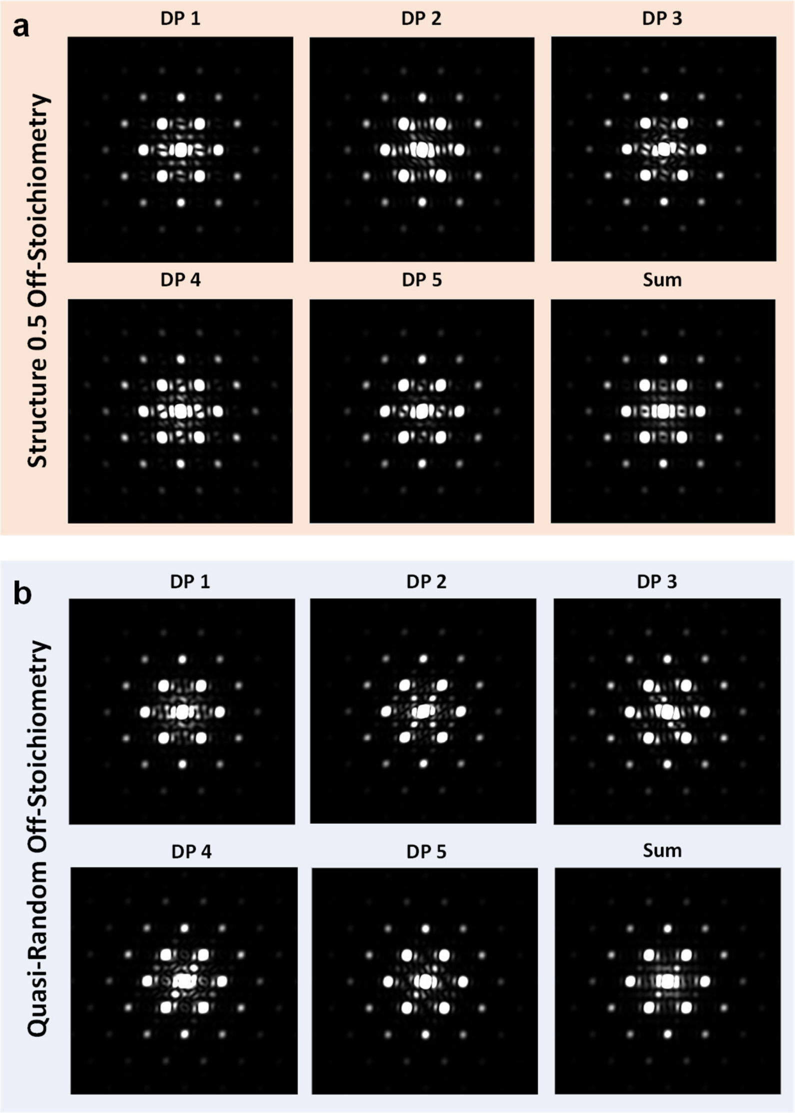

Supplementary Figure 8. Fluctuations of electron diffraction patterns among different configurations. The [110] zone axis diffraction patterns are calculated with five configurations in the off-stoichiometry Structure 0.5 models (**a**), and quasi random atomistic models (**b**) and their sum.

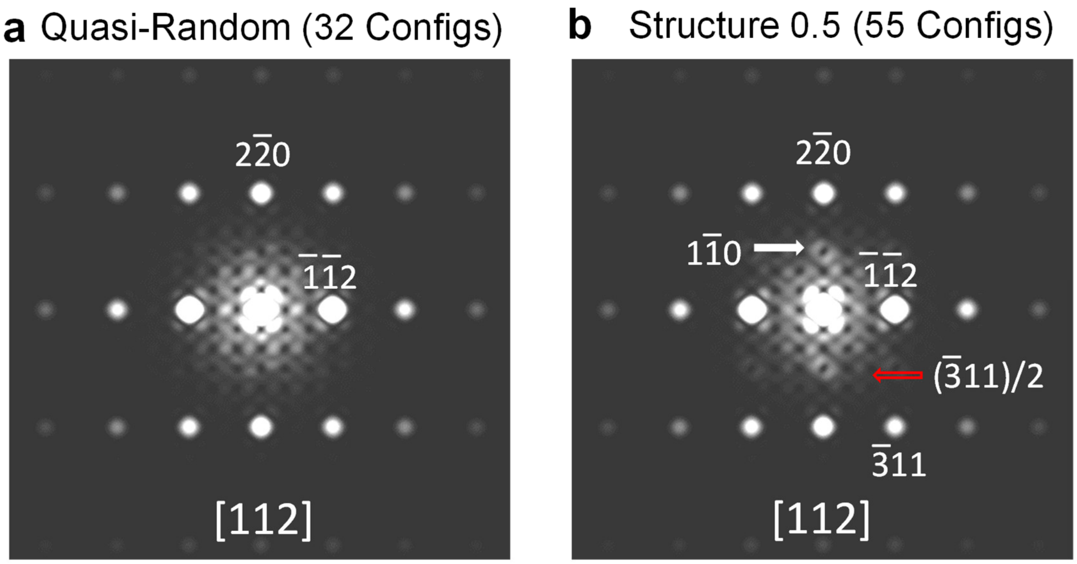

Supplementary Figure 9. Simulated electron-diffraction patterns along the [112] zone axis. Two cases are shown, one for the off-stoichiometry quasi-random models (**a**) and the other for the structure 0.5 models (**b**). The diffraction patterns are summed over multiple configurations (configs) as indicated in the figure.

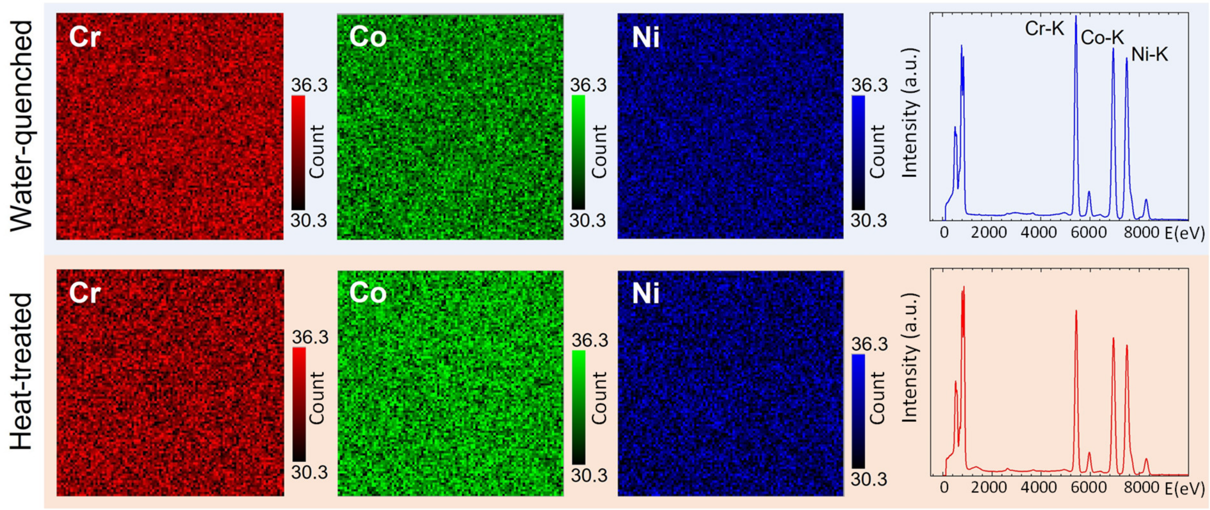

Supplementary Figure 10. Compositional uniformity in the CrCoNi samples as measured by STEM/EDS. The elemental-chemical maps obtained from a sample area of 100x100 nm^2 and the

average EDS spectra from the STEM-EDS datasets for the water-quenched and heat-treated samples.

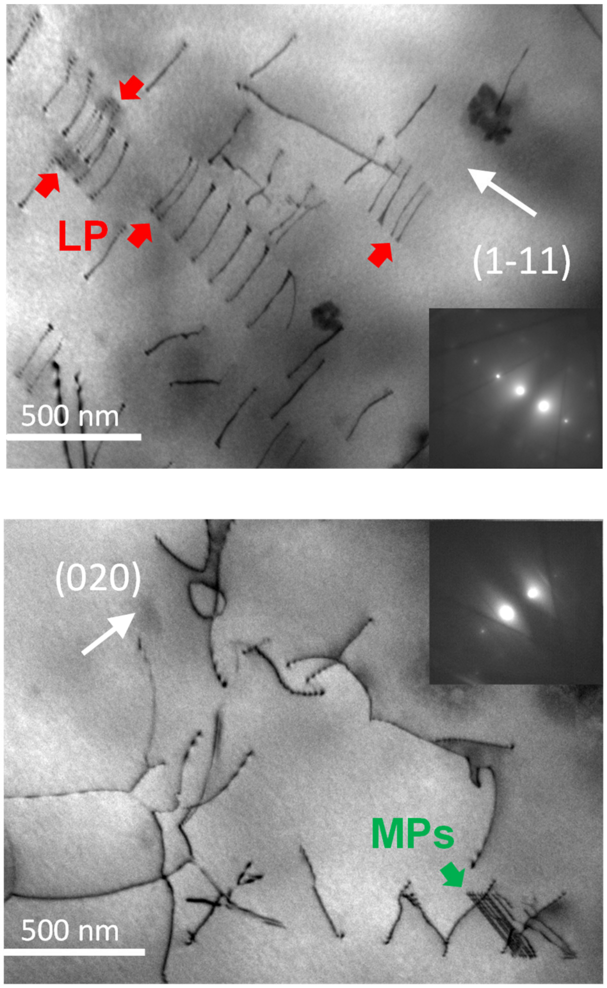

Supplementary Figure 11. Dislocation microstructure in the water-quenched CrCoNi sample. The images were recorded using the two-beam bright-field condition. These images show two additional dislocation configurations, paired dislocations (PDs), and dislocation multipoles (MPs), within the short slip trace, which are mixed with the wavy dislocation slips (**Fig. 7**, the main text) in the slightly deformed sample.

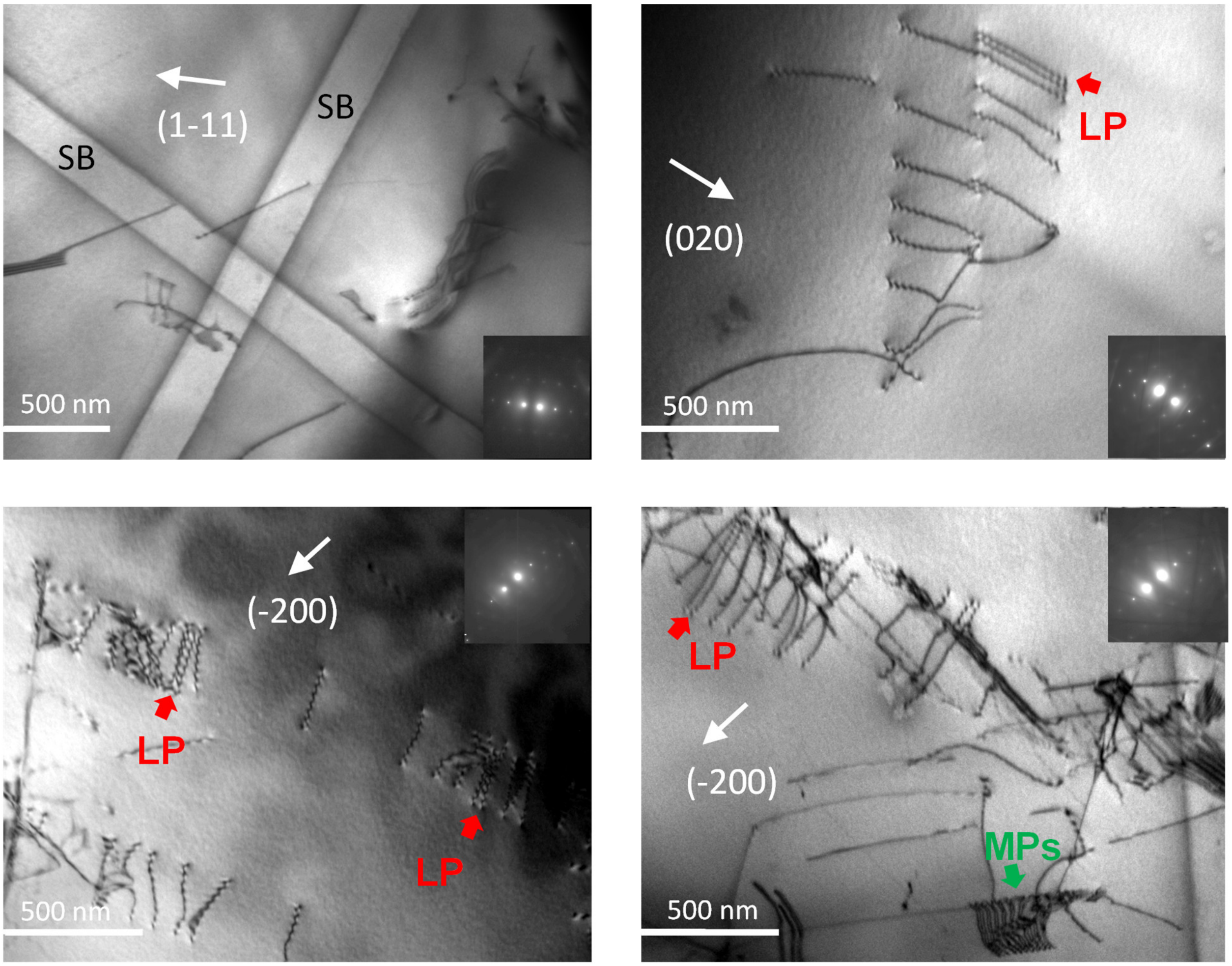

Supplementary Figure 12. Dislocation microstructure in the heat-treated CrCoNi sample. The two-beam bright-field images show long slip traces as well as two different dislocation configurations [paired dislocations (PDs) and dislocation multipoles (MPs)] within the slip traces in the slightly deformed sample.

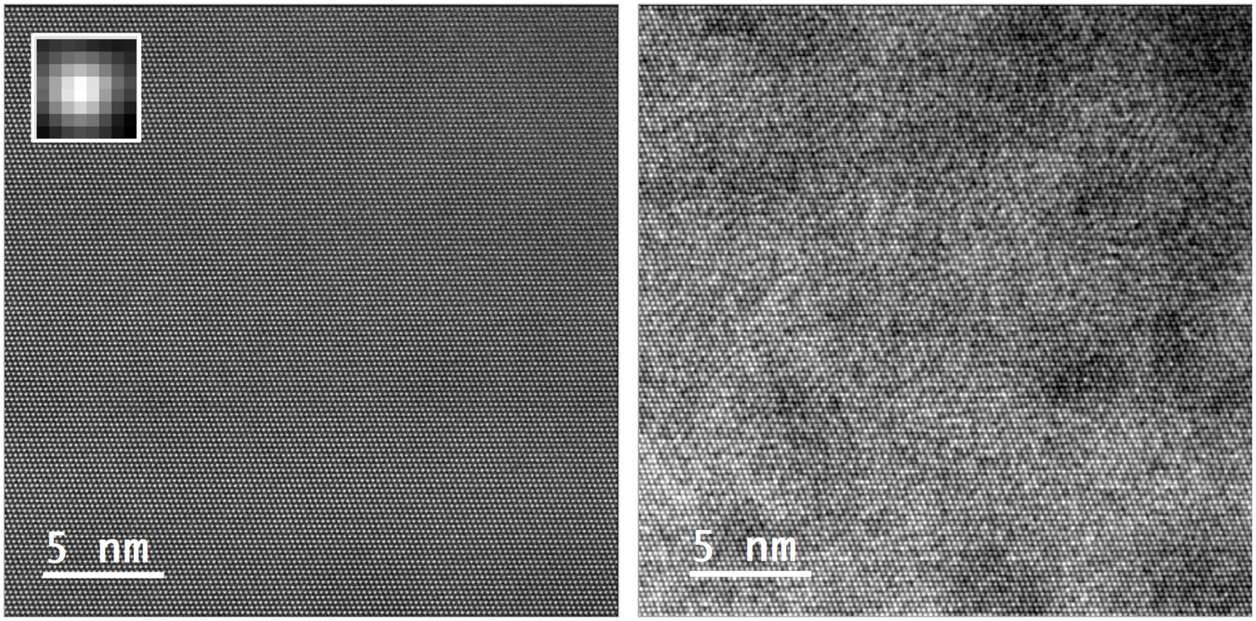

Supplementary Figure 13. Cross-correlational analysis of an atomic resolution HAADF-STEM image of Sample WQ. The left is the aligned and averaged HAADF-STEM image from a sequence of images recorded with a short dwell time. The inset is the spatially averaged template of an atomic column. The right is the cross-correlational coefficient map of the left image, obtained using template matching.

Supplemental References

1. Y.-T. Shao *et al.*, Cepstral scanning transmission electron microscopy imaging of severe lattice distortions. *Ultramicroscopy* **231**, 113252 (2021).
2. J. M. Zuo, J. C. H. Spence, *Advanced transmission electron microscopy, imaging and diffraction in nanoscience*. (Springer, New York, 2017).
3. J. Gjonnes, D. Watanabe, Dynamical diffuse scattering from magnesium oxide single crystals. *Acta Crystallographica* **21**, 297-302 (1966).
4. J.-M. Zuo, in *Springer Handbook of Microscopy,* P. W. Hawkes, J. C. H. Spence, Eds. (Springer International Publishing, Cham, 2019), chap. Electron nanodiffraction, pp. 905-969.
5. R. Zhang *et al.*, Short-range order and its impact on the CrCoNi medium-entropy alloy. *Nature* **581**, 283-287 (2020).
6. R. Yuan, J. Zhang, J.-M. Zuo, Lattice strain mapping using circular Hough transform for electron diffraction disk detection. *Ultramicroscopy* **207**, 112837 (2019).

7. F. Walsh, M. Asta, R. O. Ritchie, Magnetically driven short-range order can explain anomalous measurements in CrCoNi. *Proceedings of the National Academy of Sciences* **118**, e2020540118 (2021).
8. A. Tamm, A. Aabloo, M. Klintenberg, M. Stocks, A. Caro, Atomic-scale properties of Ni-based FCC ternary, and quaternary alloys. *Acta Materialia* **99**, 307-312 (2015).
9. J. Ding, Q. Yu, M. Asta, R. O. Ritchie, Tunable stacking fault energies by tailoring local chemical order in CrCoNi medium-entropy alloys. *Proceedings of the National Academy of Sciences* **115**, 8919 (2018).
10. L. Zhou *et al.*, Atomic-scale evidence of chemical short-range order in CrCoNi medium-entropy alloy. *Acta Materialia*, 117490 (2021).
11. J. M. Cowley, Short-Range Order and Long-Range Order Parameters. *Phys. Rev.* **138**, A1384-A1389 (1965).
12. J. M. Cowley, *Diffaction Physics*. (Elsevier Science, NL, ed. 3rd, 1995).
13. J. C. H. Spence, J. Taftø, ALCHEMI: a new technique for locating atoms in small crystals. *Journal of Microscopy* **130**, 147-154 (1983).
14. P. Lu, E. Romero, S. Lee, J. L. MacManus-Driscoll, Q. Jia, Chemical Quantification of Atomic-Scale EDS Maps under Thin Specimen Conditions. *Microscopy and Microanalysis* **20**, 1782-1790 (2014).
15. A. Guinier, G. Fournet, *Small-angle scattering of X-rays*. (Chapman & Hall, New York, 1955).
16. B. Gault, M. P. Moody, J. M. Cairney, S. P. Ringer, *Atom probe microscopy*. (Springer Science & Business Media, 2012), vol. 160.
17. D. Haley, T. Petersen, G. Barton, S. P. Ringer, Influence of field evaporation on Radial Distribution Functions in Atom Probe Tomography. *Philos. Mag.* **89**, 925-943 (2009).
18. K. Inoue, S. Yoshida, N. Tsuji, Direct observation of local chemical ordering in a few nanometer range in CoCrNi medium-entropy alloy by atom probe tomography and its impact on mechanical properties. *Physical Review Materials* **5**, 085007 (2021).
19. M. Miller, R. Forbes, Atom-probe tomography: the local electrode atom probe. Springer. (2014).
20. C. Hatzoglou *et al.*, Preferential Evaporation in Atom Probe Tomography: An Analytical Approach. *Microscopy and Microanalysis* **26**, 689-698 (2020).
21. J. Wang, P. Jiang, F. Yuan, X. Wu, Chemical medium-range order in a medium-entropy alloy. *Nature Communications* **13**, 1021 (2022).
22. A. Kumar *et al.*, Atomic-resolution electron microscopy of nanoscale local structure in lead-based relaxor ferroelectrics. *Nature Materials* **20**, 62-67 (2021).
23. X. Chen *et al.*, Direct observation of chemical short-range order in a medium-entropy alloy. *Nature* **592**, 712-716 (2021).
24. B. C. Sales *et al.*, Quantum critical behavior in the asymptotic limit of high disorder in the medium entropy alloy NiCoCr0.8. *npj Quantum Materials* **2**, 33 (2017).
25. J. M. Zuo *et al.*, Lattice and strain analysis of atomic resolution Z-contrast images based on template matching. *Ultramicroscopy* **136**, 50-60 (2014).
